# Mercy Hospital

**Published:** 1886-01

**Authors:** Edmund Andrews


MERCY HOSPITAL SURGICAL CLINIC, OF PROFESSOR EDMUND ANDREWS, M. D., LL. D.

I. FISTULA OF THE NECK.

This patient has a *fistula* in his neck, just below and behind the angle of the inferior maxillary bone. Some of the glands in this region are enlarged, but I cannot say whether it is from tubercular infiltration or some other cause, either malignant or

*The patches were entirely healed in three weeks after the operation, but a few spots will require to be scraped again.

non-malignant. This abscess has been on the patient's neck for about a year. Probing reveals the presence of a sinus leading inwards and downwards to several enlarged cervical lymphatic glands; we will now anæsthetize the patient, and divide, with a probe-pointed bistoury, the tissues forming the *fistula*. There are two tuberculous glands present in this abscess. These must be removed to secure recovery, and even then, it cannot always be promised, for, no matter how careful the surgeon may be, sometimes a new abscess will form. In a large majority of instances the operator will be quite successful in eradicating the tubercular disease in the region, by enucleating all of the infiltrated glands he can discover. I have now extirpated the caseous glands, and, with my finger in the *fistula*, I can feel the carotid artery pulsating. This operation has taken a little more time than it would under ordinary circumstances, but, on account of the close proximity of the carotid artery, and to avoid wounding it, I have had to work unusually slowly and carefully. I will not introduce the drainage tube, because it would lie directly on the artery mentioned, and, by the constant pulsation of the vessel against the rubber drainage-tube, the continuity of the arterial walls would, possibly, be injured, though it is by no means certain that such would be the case. In abscesses similar to this, with a large artery near the surface, drainage is best facilitated by the use of silk setons. The etiology, prophylaxis and treatment of these tubercular glands have not been reduced to a science; if they were, it would be much better for the patient. In cases of *sarcoma, carcinoma* or *tuberculosis* of any of the superficial lymphatic glands, the lungs, also, are apt to become infiltrated by the disease, the germs being carried to the lungs by the venous system. It is for this reason that surgeons consider it the best treatment to excise these diseased glands. As proof that the lungs are prone to tubercular infection when the lymphatics are previously affected by a caseous deposition, I will relate the history of a case of glandular enlargement in the axillary region, which before the incision was supposed to be limited to only one or two glands, but after cutting into them many were found to be involved, some twenty or more were removed, and, instead of being entirely superficially located, as was at first supposed, some were found to be very close to the axillary artery. This patient then went east, to Montreal, a peculiarly tubercular climate, at least more so than Chicago. After having been there sometime, a prominent physician, who was eminently able, diagnosed incipient pulmonary *tuberculosis.* The patient was then sent to Colorado, where he in part or almost wholly recovered. *A propos* of this affection, as it is exceedingly interesting, I will mention a patient of mine who had *tuberculosis* of both *testes;* he did not want them extirpated; I did not remove them, and, for a period of twenty years, I have rather defied authorities by not excising these caseous testicles. My treatment has been to lance them occasionally and thus, theoretically, to save them; I have never heard of any of my patients thus treated being affected with *tuberculosis pulmonalis.* Several eastern surgeons agree with me in thinking that there is too much indiscriminate castration as a remedial agency in tubercular *testes.* The patient before us gives a history of *phthsisis,* his father having died of the affection. There is no other history of *phthisis pulmonalis* in his family. The silk setons being in place, the wound will be stitched by a continued suture and dressed antiseptically.

### II.—CHRONIC SYNOVITIS OF THE LEFT KNEE.

This case of synovitis has continued through a period of three years, and the patient thinks it is a result of having had to kneel at his work for a period of two weeks. Eight months ago, however, a horse kicked him just below the patella, and the disease has been growing gradually worse since. In a large number of cases of synovitis, the patients can recall some act of violence that the part has received; but, even then, the blow is not always the cause of the inflammatory process, for tuberculosis figures very prominently as an etiological factor in the production of synovial inflammations. This patient was born in Marshall County, Indiana, a not specially tubercular region, if anything more favorable than Chicago. I mention his birthplace, because, if he came from a peculiarly tuberculous climate, we should apprehend that the synovitis would be very apt to be complicated by a caseous deposition in the tissues of the joint. As it is, we do not so much fear it. Inspection does not reveal the hollow that is usually found on each side of the patella. There is no anchylosis of the joint. It permits slight motion without pain. There is too much synovia present. The patient knows comparatively little about the anatomy of the part, but knows enough about the nervous impressions, for such patients usually hold the knee bent at a certain angle, as, in any other position, it is very painful. By this protracted flexion, the muscles, as well as the ligaments, attain a permanent contraction, thus hindering movement. In this case there is an inflammation of all the tissues of the joint; beginning with the synovial membrane, and going down through the bone, involving even the canaliculi. Towards the surface we find the muscles and ligaments are also involved in the inflammatory process. The slightest pressure of the synovial surfaces against each other produces considerable pain; but even a large amount of stretching does not elicit pain. Treatment: Cases of synovitis, either tubercular or non-tubercular, would tend to a spontaneous recovery more readily, if it were not for the constant friction and pressure to which most patients subject their inflamed knees. Some surgeons think that friction is the only element to be eliminated, and accordingly, secure immobility to effect a recovery. Friction and pressure of the joint-surfaces upon each other are both evil influences, and both must be overcome by the combined help of extension and immobility. In all cases of synovitis except those of rheumatic origin, extension is an important part of the treatment. In the first place Sayre's apparatus, or any simple splint, using rubber adhesive plaster to retain the appliance *in situ* is good treatment. Sayre claims that an effective extension-splint cannot be applied to a bent knee until it is straightened. It is true, his brace is not applicable to bent articulations, but other appliances are perfectly effectual and there is no real difficulty in their use. First, the patient can be kept in bed, and the limb be flexed by putting it over a double inclined plane or a pile of pillows, and applying adhesive-plasters and weight and pulley. By gradually lowering the angle, the limb will be both straightened and kept extended. Secondly, a splint can be arranged in the form of a bent trough, of light tin or brass, fitted to the posterior half of the member, and secured by leather straps laced to the thigh, and for extension, by adhesive straps attachedto the legs, extension downward may be made. Thus extension and immobility in the bent position are secured. Sayre's knee-splint is not used advantageously when the patient cannot be seen very often. If not watched, the patient is apt to mismanage the dressing and even to create ulcers on the limb, by the pressure of the edges of the metal supports, whose position he does not know how to control. I like the bent splint above mentioned. It is constructed in the following manner. Take a plaster cast of the posterior half of the leg and thigh, and have a tinsmith fit a brass or tin casing to it. Above the knee rivet on in front a pair of firm leather flaps to be laced up and thus surround the thigh for counter-extension. The ben, knee will now fit nicely into the splint. Apply a broad adhesive strap on each side of the leg, fasten to the lower end of each strap a piece of strong elastic webbing. Let the webbing run down to friction rollers at the bottom of the splint, then turn it across the rollers, and upward outside of the apparatus and fasten to it a pair of button-knobs, or two buckles. The tightening of these straps makes firm and motionless extension.

